# Supplementary figures and images for: Investigation and Expression Analysis of R2R3-MYBs and Anthocyanin Biosynthesis-Related Genes during Seed Color Development of Common Bean (Phaseolus vulgaris)
Source: Plants (Basel). 2022 Dec 5;11(23):3386. doi: 10.3390/plants11233386 (PMC9736660; doi:10.3390/plants11233386)

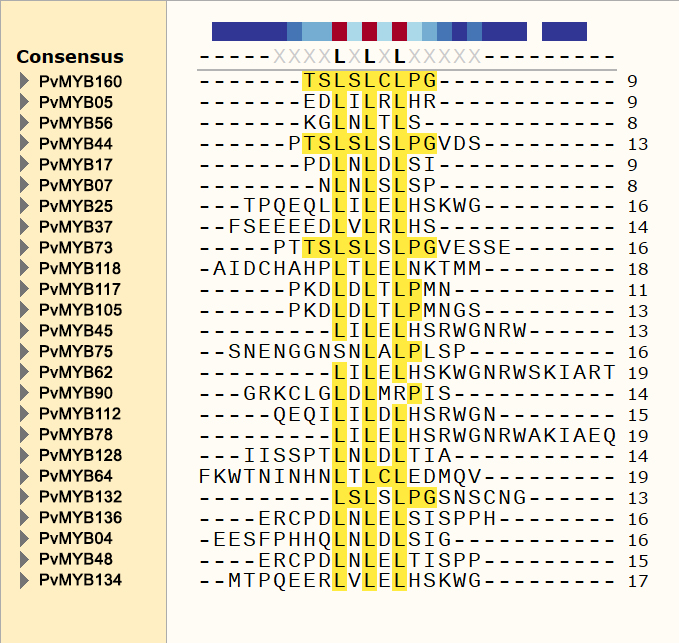

Supplement: Supplementary file 1 [file plants-11-03386-s001.zip › Figure S1.jpg]

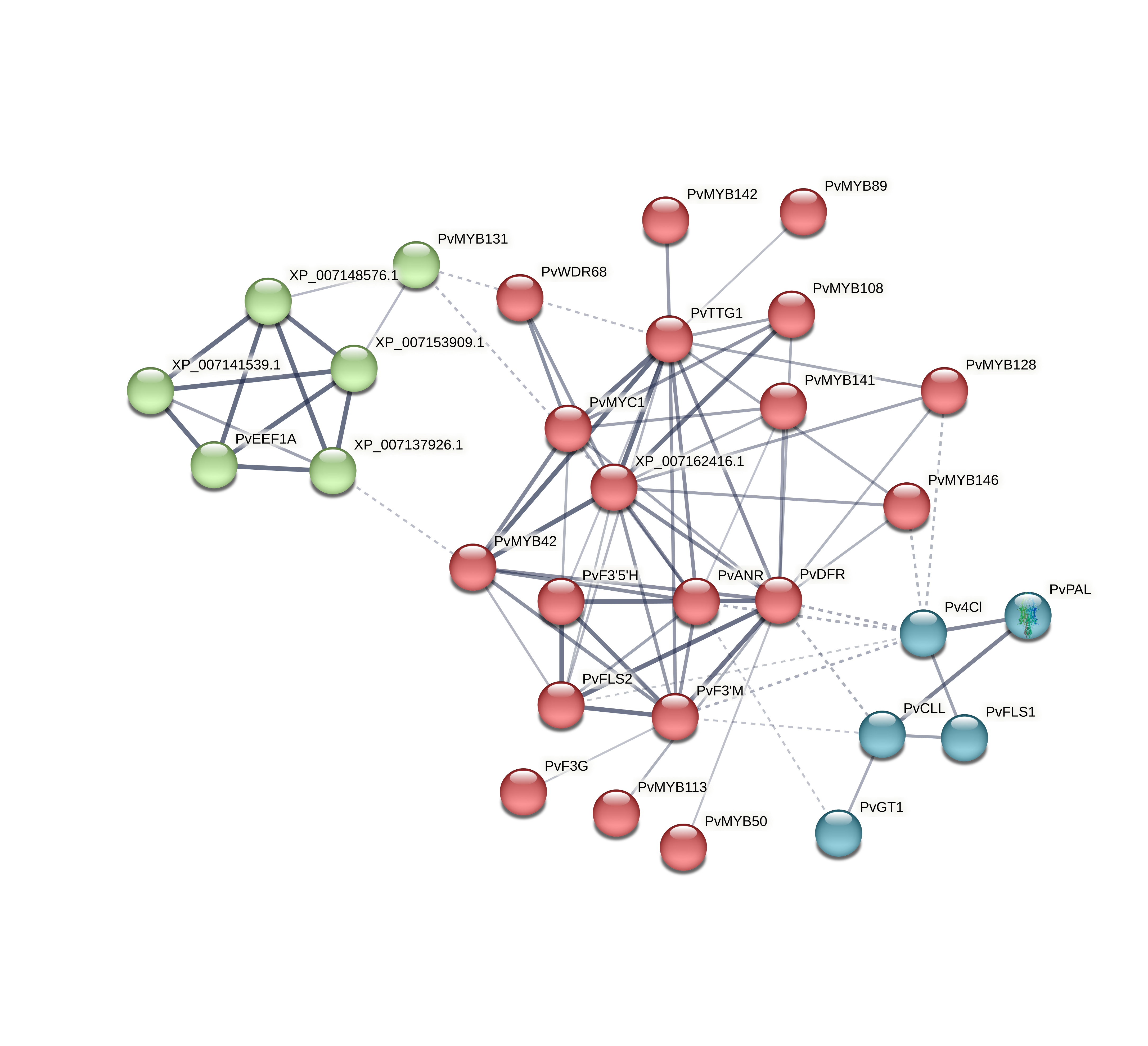

Supplement: Supplementary file 1 [file plants-11-03386-s001.zip › Figure S3.png]

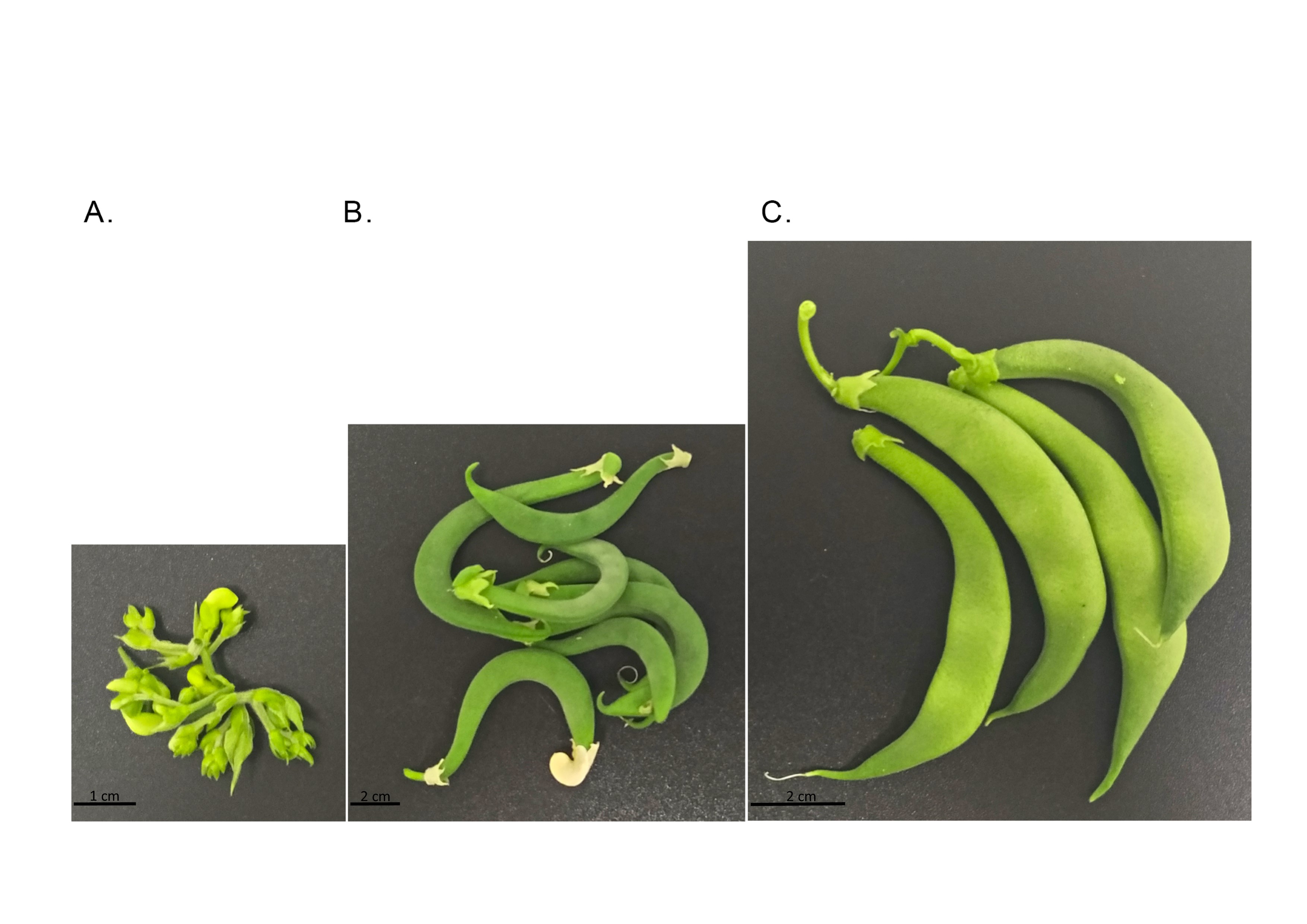

Supplement: Supplementary file 1 [file plants-11-03386-s001.zip › Figure S4.jpg]
